# Fibulin-6 regulates pro-fibrotic TGF-β responses in neonatal mouse ventricular cardiac fibroblasts

**DOI:** 10.1038/srep42725

**Published:** 2017-02-17

**Authors:** Arpita Chowdhury, Lisa Hasselbach, Frank Echtermeyer, Nidhi Jyotsana, Gregor Theilmeier, Christine Herzog

**Affiliations:** 1 Department of Anesthesiology and Intensive Care Medicine, Hannover Medical School, Hannover, Germany; 2 Department of Cellular Biochemistry, University Medical Center Göttingen, Göttingen, Germany; 3 Department of Hematology, Hemostasis, Oncology and Stem cell Transplantation, Hannover Medical School, Hannover, Germany; 4 Perioperative Inflammation and Infection, Department of Human Medicine, Faculty of Medicine and Health Sciences, University of Oldenburg, Oldenburg, Germany

## Abstract

Fibulin-6, an essential component of extracellular matrix determines the architecture of cellular junctions in tissues undergoing strain. Increased expression and deposition of fibulin-6 facilitates fibroblast migration in response to TGF-β, following myocardial infarction in mouse heart. The underlying mechanism still remains elusive. In conjunction with our previous study, we have now demonstrated that in fibulin-6 knockdown (KD) fibroblasts, not only TGF-β dependent migration, but also stress fiber formation, cellular networking and subsequently fibroblast wound contraction is almost abrogated. SMAD dependent TGF-β pathway shows ~75% decreased translocation of R-SMAD and co-SMAD into the nucleus upon fibulin-6 KD. Consequently, SMAD dependent pro-fibrotic gene expression is considerably down regulated to basal levels both in mRNA and protein. Also, investigating the non-SMAD pathways we observed a constitutive increase in pERK-levels in fibulin-6 KD fibroblast compared to control, but no change was seen in pAKT. Immunoprecipitation studies revealed 60% reduced interaction of TGF-β receptor II and I (TGFRII and I) accompanied by diminished phosphorylation of TGFRI at serin165 in fibulin-6 KD cells. In conclusion, fibulin-6 plays an important role in regulating TGF-β mediated responses, by modulating TGF-β receptor dimerization and activation to further trigger downstream pathways.

Myocardial remodeling (MR) and fibrosis leading to cardiac failure is largely characterized by the pronounced induction and activation of TGF-β. It has been referred to act as a ‘master switch’ in the pathogenesis of various experimental animal models of myocardial infarction (MI) and cardiac failure as well as in human hypertrophic and ischemic cardiomyopathy^1^,^2^,^3^,^4^. Extra Cellular Matrix (ECM) critically regulates and in turn is regulated by TGF-β. Various studies have shown that TGF-β is crucial in ECM deposition and metabolism; reciprocally ECM modulates TGF-β sequestration, activation and signaling^5^,^6^,^7^.

Many ECM proteins like fibrillin^8^, fibronectin^9^ and decorin^10^ are pivotal for TGF-β activity. However, over the past years fibulins, a family of secreted ECM glycoproteins, have also been described to play an important role in regulating TGF-β. Fibulin-4 has been shown to bind latent TGF-β binding protein-1 and fibrillin-1 thus regulating the activation of TGF-β^11^. Similarly fibulin-5 is reported to bind latent TGF-β binding protein-2, which in turn regulates elastic fiber assembly^12^. Studies in a knockout mouse model for fibulin-2 depicted marked reduction in TGF-β mediated effects after MI or angiotensin II induced cardiac hypertrophy^13^. However the underlying mechanisms have not been explored extensively.

The emerging role of fibulins in TGF-β regulation and MR is an important gateway to understand the mechanisms governing the process. Fibulin-6, the largest member of the fibulin family has been characterized in the process of MR by our group^14^. It is known for its role in the formation of transient cell contacts during tissue organization, cell migration, basement membrane invasion as well as in the formation of stable cell-cell and cell-ECM contacts, mainly in epithelial tissues of zebra fish and *c. elegans*^15^,^16^,^17^. Due to the fact that fibulin-6 knockout is lethal at the blastocyst stage^18^ the functional role of fibulin-6 in the mammalian system is poorly understood. However, some proteomic and transcriptomic studies in MI and MI/R models of mouse and pig, suggested an involvement of fibulin-6 in the process of remodeling^19^,^20^. Previously we have shown an increased expression of fibulin-6 during MR in a murine model of MI with a similar expression pattern in human failing heart. Furthermore we showed that fibulin-6 is abundantly expressed and deposited in ECM produced by cardiac fibroblasts, which in turn influences fibroblast migration in a TGF-β dependent manner^14^.

In our present study we have further explored the role of fibulin-6 on TGF-β mediated stress fiber formation and fibroblast contraction, which are the basic phenomenon for wound healing. To understand the mechanistic role of fibulin-6 during MR, we analyzed fibulin-6 regulated TGF-β signaling *in vitro* by using neonatal mouse ventricular cardiac fibroblasts (nCF).

## Results

### Absence of fibulin-6 inhibits stress fiber formation upon TGF-β stimulation

**Figure 1:**
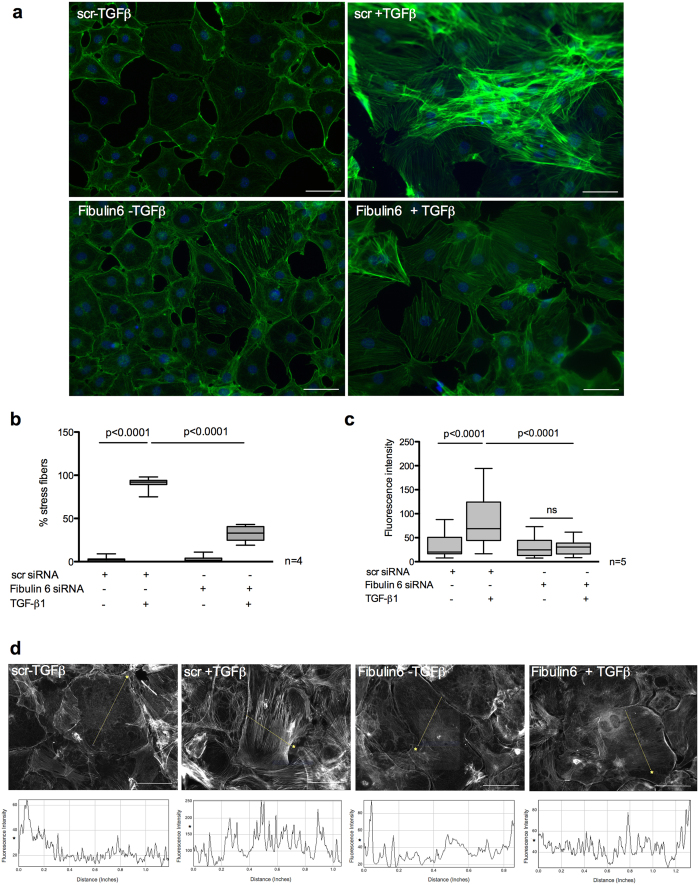
Attenuated stress fiber formation in fibulin-6 KD cells after TGF-β stimulation. Fibroblasts isolated from neonatal mouse hearts and stimulated with TGF-β were stained with fluorescently labelled phalloidin for stress fibers. (**a**) scr-siRNA transfected nCF display increased stress fiber formation after TGF-β stimulation compared to non stimulated cells. In fibulin-6 KD cells stress fiber formation after TGF-β stimulation is almost absent (scale bar = 100 μm). (**b**) Total % of cells expressing stress fibers normalized to total number of cells. (n = 4, p < 0.0001) (**c**) Quantitative analysis of fluorescence intensity displayed by the stress fibers in the representative cells shown in panel (**d**) where each peak value correspond to 3 regions of the same cell with 6 cells analyzed per picture (n = 5, p < 0.0001). Differences in the mean value between groups are determined by two tailed, non-parametric Mann-Whitney U test. (**d**) Fluorescence intensities of the line drawn across the cell shown in corresponding panels below were quantified using Image J software. Asterisks demarcate cells being quantified and their corresponding line graphs below.

In our previous report we have demonstrated that in the absence of fibulin-6 differentiation of fibroblasts into myofibroblats upon TGF-β stimulation is abolished. This was evidenced by substantial downregulation of α-smooth muscle actin (αSMA) expression both at mRNA and protein level in fibulin-6 KD cells^14^, which otherwise would incorporate into stress fibres during the process of myofibroblast differentiation^21^. TGF-β is known to mediate the induction of the scattered actin microfilaments into organized stress fiber bundles^22^. These actin stress fibers furnish increased contractility to fibroblasts when anchored to focal adhesions, which connect the ECM to the actin cytoskeleton^23^. This is clearly observed by phalloidin staining of control fibroblasts exposed to TGF-β, where the formation of stress fibers is evident. However, fibulin-6 KD fibroblasts barely formed stress fibers upon TGF-β stimulation (Fig. 1a). We assessed the number of cells expressing stress fibers normalized to total cell number and observed a decrease in stress fiber formation in fibulin-6 KD cells by 65% (Fig. 1b). A quantitative measurement of stress fiber density by incorporating line profiles across the cytoplasm that identified stress fibers by their increased fluorescence relative to areas devoid of stress fibers, revealed 2.5-fold increased peak values for TGF-β induced scrambled (scr) siRNA transfected nCF, whereas peaks for fibulin-6 KD cells displayed a negligible increase upon stimulation (Fig. 1c and d). To analyze whether fibulin-6 KD shows not only an effect on differentiation but also on cell proliferation we did a cell proliferation assay with fibulin-6 KD- compared to scr-transfected fibroblasts but observed no differences in cell proliferation over a time period of 48 hours (Suppl. Fig. 1). Next, we asked whether the effect of fibulin-6 on cytoskeletal rearrangements is specific for the TGF-β dependent pathway or whether signaling by other profibrotic agonists is also affected by fibulin-6. Treatment with lysophosphatidic acid (LPA) and TNF-α induces the formation of F-actin, which is observed mostly at the cellular cortex. These treatments did not induce stress filament formation across the cells unlike TGF-β1 treatment. However, we did not observe any substantial differences in fibulin-6 KD cells compared to scr–transfected fibroblasts (Suppl. Fig. 2), which indicates that the effect of fibulin-6 on stress fiber formation is specific for the TGF-β signaling pathway.

### Fibulin-6 is important for TGF-β mediated wound contraction

**Figure 2:**
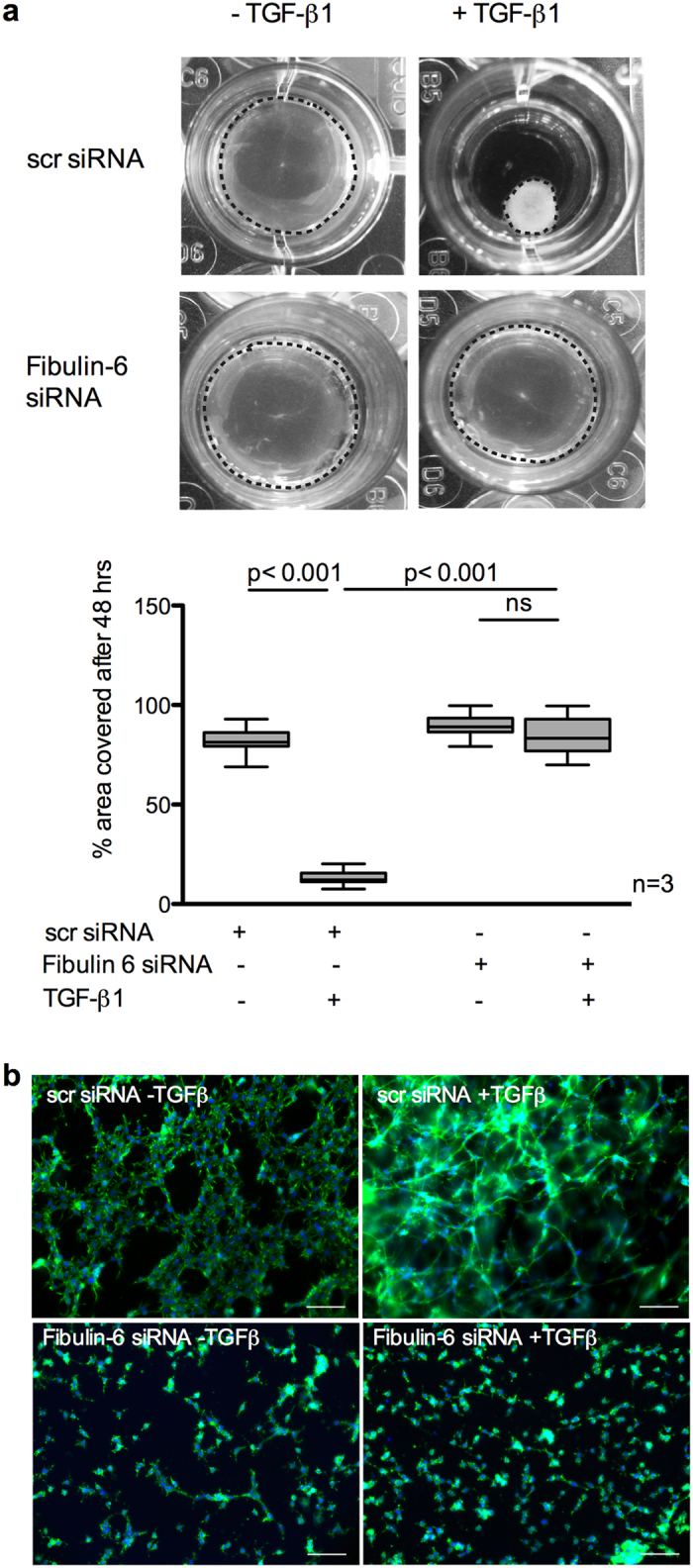
TGF-β mediated collagen contraction is reduced in fibulin-6 KD CF. Collagen gels carrying either scr-siRNA or fibulin-6 KD 3T3 CF were exposed to TGF-β to induce contraction. (**a**) A representative picture of collagen gels 48 h after TGF-β stimulation is shown. The area of the gels (marked by the dotted circle around the collagen gel) before and after stimulation was measured using Image J and is represented in the corresponding graph. After TGF-β stimulation the size of collagen gels carrying control cells is significantly reduced while collagen gels carrying fibulin-6 KD cells display no contraction (n = 3, p < 0.001, nonparametric Mann-Whitney U test). The experiment was repeated three times in quadruplicates. (**b**) Photographs of collagen gels stained for phalloidin after 48 hours of TGF-β stimulation (scale bar = 100 μm). In scr-siRNA transfected 3T3 CF the formation of cellular networks during contraction can be observed, while actin cytoskeletal network and cell-cell contact in fibulin-KD CF are absent.

To test whether impaired stress fiber formation in fibulin-6 KD nCF affects wound contraction, we used an *in vitro* collagen gel contraction assay. When stimulated with TGF-β the collagen gels loaded with nCF showed 84% contraction while collagen gels with fibulin-6 KD fibroblasts showed negligible contraction (Fig. 2a) upon TGF-β stimulation. Within these collagen gels actin stress fibers were stained with phalloidin to visualize the formation of cellular networks that are responsible for collagen gel contraction in TGF-β stimulated control cells. Staining of TGF-β stimulated control fibroblasts in collagen gels revealed that stress fibers of individual fibroblasts are connected by cell-cell contacts and form an actin cytoskeleton network (Fig. 2b). In contrast, in fibulin-6 KD fibroblast populated gels stress fibers are missing and cells are not connected which finally results in arrested contraction of the gels.

### Fibulin-6 knockdown fibroblasts express less pro-fibrotic genes

**Figure 3:**
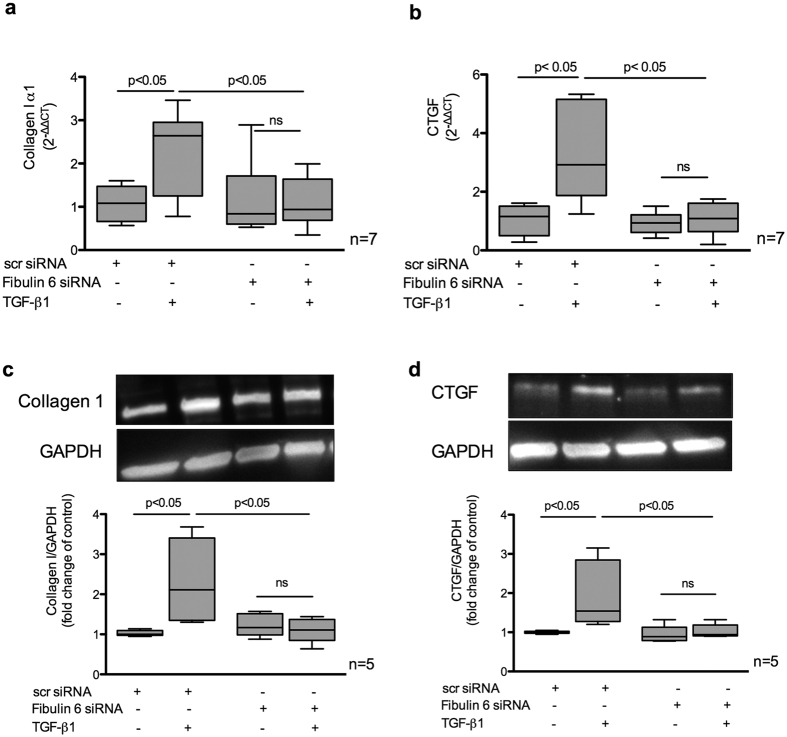
Pro-fibrotic gene expression in TGF-β induced fibroblasts. nCF transfected with scr siRNA or Fibulin-6 siRNA, after being exposed to TGF-β were subjected to RNA or protein extraction for real-time PCR or western blotting respectively. (**a**) Collagen I α1 and (**b**) CTGF mRNA expression is increased after TGF-β stimulation in scr- but not in fibulin-6-siRNA transfected nCF (n = 7, p < 0.05, nonparametric Mann-Whitney U test). Real-time RT-PCR signals were normalized to hypoxanthine phosphoribosyl-transferase (HPRT) gene expression and data are expressed as 2^−ΔΔCT^. (**c** and **d**) Densitometric analysis of western blots revealed upregulation of (**c**) collagen I α1 and (**d**) CTGF upon TGF-β stimulation in scr-siRNA transfected nCF. In fibulin-KD nCF no upregulation could be observed (n = 5, p < 0.05, nonparametric Mann-Whitney U test).

TGF-β is a known fibrotic cytokine responsible for regulating various pro-fibrotic genes^24^. We find that in the absence of fibulin-6, TGF-β stimulation in nCF could not induce the expression of Collagen I and CTGF at the transcriptional level (Fig. 3a and b) as well as at the protein level (Fig. 3c and d).

### SMAD signaling is disrupted in Fibulin-6 deficient fibroblasts

**Figure 4:**
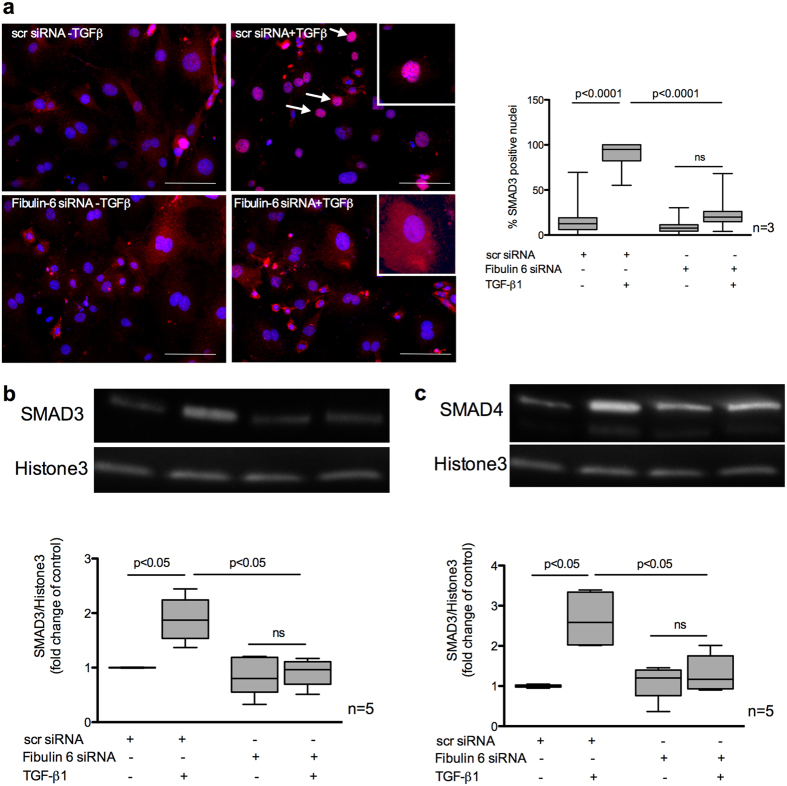
Nuclear localization of SMAD3/4 in TGF-β stimulated CF. nCF transfected with scr siRNA or Fibulin-6 siRNA, with and without TGF-β stimulation are fixed and stained for SMAD3 expression or are processed for nuclear lysate preparation. (**a**) Cells are stained with SMAD3 (red) and DAPI (blue) (Scale bar = 100 μm). In scr siRNA transfected CF translocation of SMAD3 into the nucleus can be observed upon TGF-β stimulation. Arrows indicate SMAD3 positive nuclei. In fibulin-6 KD CF SMAD3 staining is almost absent in nuclei. High magnification inserts show nuclear SMAD3 staining in scr siRNA transfected CF and cytoplasmic SMAD3 staining in fibulin-6 siRNA transfected CF. A corresponding graph shows quantitative measurement of SMAD3 positive nuclei compared to the total number of cells (n = 3, p < 0.0001, nonparametric Mann-Whitney U test). The experiment was repeated three times with 10 pictures analyzed each time. Western blot showing (**b**) SMAD3 and (**c**) SMAD4 nuclear signal in TGF-β stimulated nuclear lysates compared to the non-stimulated set. Westerm blot and corresponding densitometric analysis show increased localization of (**b**) SMAD3 and (**c**) SMAD4 in nuclear extracts after TGF-β stimulation, which is absent in fibulin-6 KD CF (n = 5, p < 0.05 nonparametric Mann-Whitney U test).

After binding of TGF-β to its specific cognate cell surface receptors, it predominantly induces the canonical SMAD dependent signaling pathway. This in turn is responsible for manifesting various pro-fibrotic effects^25^. Upon TGF-β binding to TGFRs the receptor-SMADs (R-SMAD) 2 and 3 are phosphorylated and form a complex with co-SMAD (SMAD4). This complex translocates into the nucleus where it binds to its transcriptional promoter to initiate transcription of various genes required during MR like αSMA, collagen I or CTGF (see also Suppl. Fig. 3). Thus, to answer whether the disrupted response of TGF-β in fibulin-6 KD cells is due to the disrupted SMAD signaling or not, we looked into SMAD’s nuclear localization upon TGF-β stimulation. We observed 75% less nuclear localization of SMAD3 in fibulin-6 KD- compared to control-fibroblasts (Fig. 4a). Western blot analysis of SMAD3 and SMAD4 in nuclear extracts of TGF-β stimulated cells showed elevated expression in scr-siRNA transfected cells, while fibulin-6 KD cells displayed diminished nuclear localization upon TGF-β stimulation (Fig. 4b and c).

### Fibulin-6 KD affects the TGF-β mediated ERK- but not PI3K/AKT-pathway

**Figure 5:**
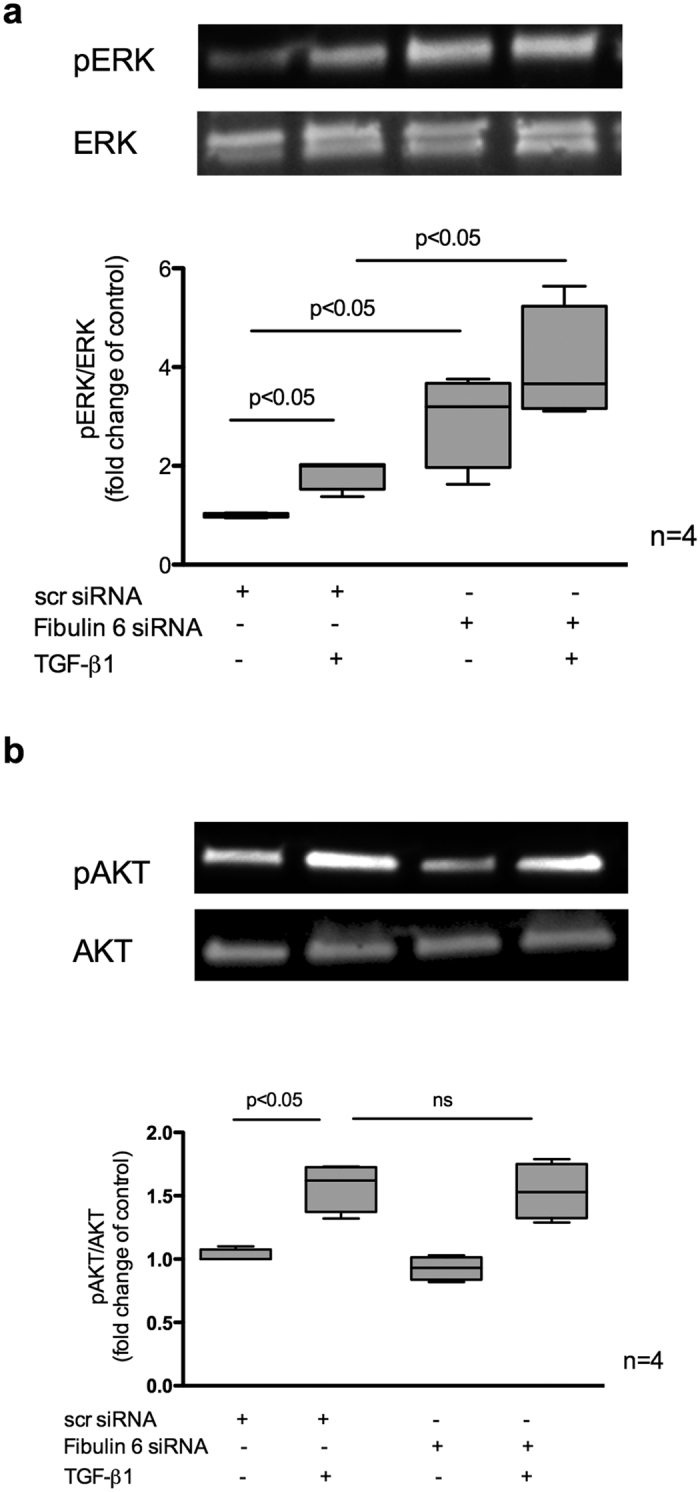
Effect of fibulin-6 KD on Non-SMAD pathways. Fibulin-6 siRNA and scr-siRNA transfected cells with and without TGF-β treatment for 24 h are processed for WB analysis. (**a**) pERK compared to total ERK, which represents a constitutive increase upon KD of fibulin-6 in comparison to control cells (n = 4, p < 0.05, nonparametric Mann-Whitney U test) and (**b**) pAKT compared to total AKT (n = 4, p < 0.05, nonparametric Mann-Whitney U test), which doesn’t show any significant increase upon TGF-β induction in KD cells compared to control.

Apart from the canonical SMAD signaling pathway, TGF-β1 also directly activates certain non-canonical pathways to reinforce, attenuate or modulate downstream cellular responses. To investigate whether fibulin-6 affects the non-SMAD pathways as well or not, we looked at the TGF-β induced ERK as well as PI3K/AKT phosphorylation status. Interestingly, we observed that compared to scr-transfected nCF, ERK pathway was already activated in fibulin-6 KD cells, which is evident from a significant 30% increase (p < 0.05) in pERK normalized to total ERK (Fig. 5a). TGF-β stimulation in control nCF increased pERK levels up to 2-fold indicating an active Ras-Raf-MAPK-ERK pathway, which was more pronounced in fibulin-6 KD cells. We also analyzed the levels of phosphorylated AKT depicting the activated state of the pathway. We observed a significant increase of pAKT (p < 0.05) upon TGF-β induction with no difference between control and fibulin-6 KD nCF (Fig. 5b).

### Fibulin-6 is involved in receptor-heterodimerization of TGF-β receptors and phosphorylation of TGFRI

**Figure 6:**
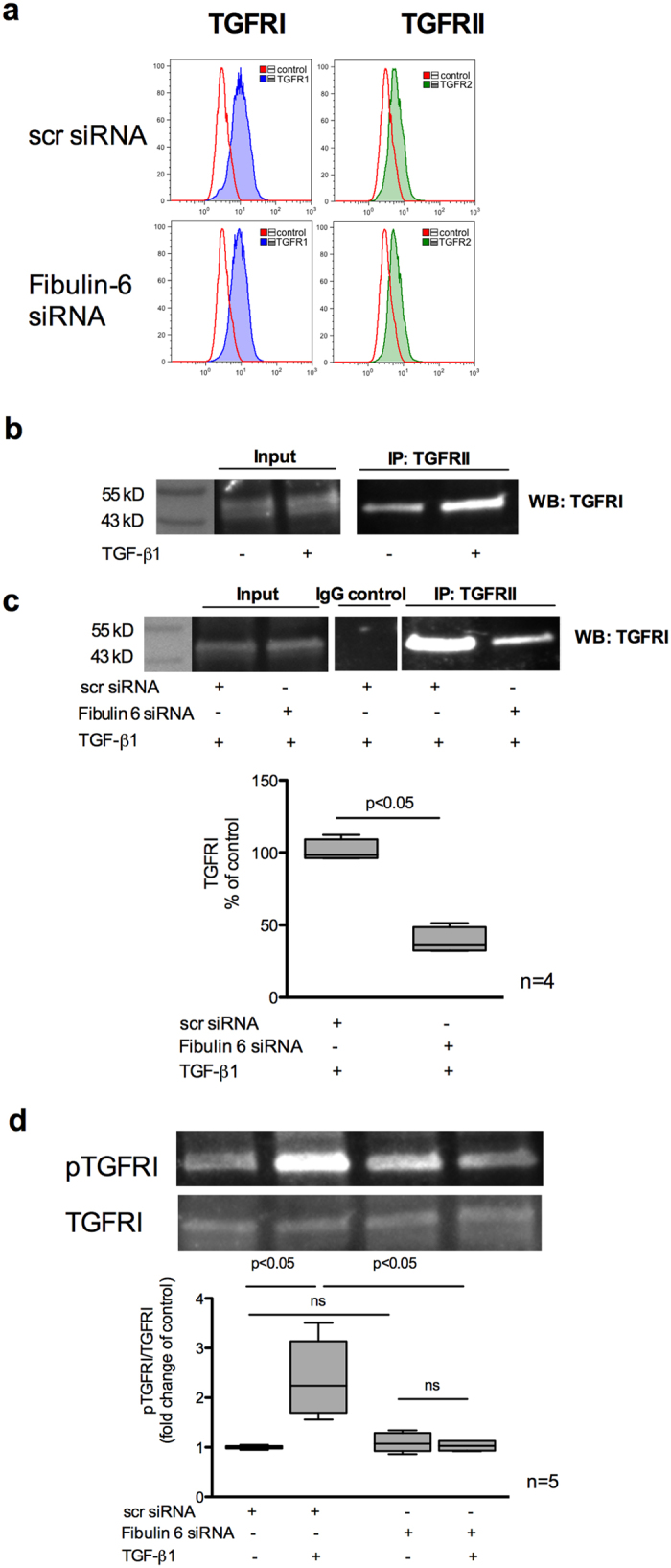
TGFRI and TGFRII association in fibulin-6 KD cells upon TGF-β stimulus. scr siRNA or fibulin-6 siRNA transfected nCF, after TGF-β stimulation are subjected to (**a**) FACS analysis. No difference in the amount of TGFβRI and TGFβRII on the surface of control and fibulin-6 KD cells was observed. (**b**) Immuno-precipitation of TGFRII from cell lysates of scr transfected and TGF-β stimulated cells, shows more accumulation of TGFRI after western blotting compared to non TGF-β treated nCF. (**c**) Immuno-precipitation of TGFRII followed by WB for TGFRI from cell lysates of scr transfected or fibulin-6 KD cells after TGF-β stimulation display reduced association of receptors in fibulin-6 KD condition. The control input lanes shows no difference and IgG control is also clean (n = 4, p < 0.05). (**d**) Phosphorylation status of TGFRI was analyzed by western blot using phospho-TGFRI specific antibody. Densitometric analysis of western blot display decreased phosphorylation of TGFRI in fibulin-6 KD cells after TGF-β stimulation compared to control cells (n = 5, p < 0.01, nonparametric Mann-Whitney U test).

Next we aimed to elucidate the possible underlying mechanisms on how fibulin-6 actually interacts with the TGF-β signaling pathway. We previously showed that fibulin-6 deposited in the matrix is sufficient for TGF-β induced stress fiber formation in nCF^14^, which indicates that fibulin-6 possibly acts as an external factor to affect TGF-β signaling. TGF-β signaling is triggered at the cell surface when any of the TGF-β superfamily ligands bind to TGFR II. Receptor-heterodimerization of TGFRI and TGFRII followed by phosphorylation of TGFRI at serine165^26^ represent the down stream signal, which is a crucial step for further phosphorylation and activation of R-SMADs. Thus, we first tested the impact of fibulin-6 on the presentation of TGFRs on the cell surface. FACS analysis revealed that the amount of TGFRI and TGFRII on the cell surface is not affected by fibulin-6 (Fig. 6a). Secondly, we examined the receptor-dimerization of TGFRI and TGFRII when stimulated with TGF-β. A co-immunoprecipitation experiment for TGFRI with TGFRII in scr-transfected nCF displayed an increased interaction between the two receptors after TGF-β stimulation compared to non-treated cells (Fig. 6b), while the input lanes showed no difference. Further we observed 60% reduced interaction of TGFRI with TGFRII in TGF-β stimulated fibulin-6 KD nCF as compared to control cells (Fig. 6c). To finally proof that fibulin-6 plays a critical role in TGFR mediated signaling we analyzed phosphorylation of TGFRI at Ser165 and observed 2.4-fold increase of phosphorylated TGFRI in TGF-β stimulated fibroblasts, while receptor phosphorylation in fibulin-6 KD fibroblasts after stimulus was equivalent to non-stimulated cells (Fig. 6d).

## Discussion

In this study we demonstrate that the ECM protein fibulin-6 is a notable regulator of TGF-β signaling in neonatal cardiac fibroblasts. Previously, we reported that fibulin-6 is predominantly produced by fibroblasts rather than myofibroblasts and is deposited in the cardiac ECM, particularly when the tissue is experiencing significant mechanical strain^14^. A similar observation *in vitro* is evidenced after CFs are exposed to TGF-β. Besides, when fibroblasts and their surrounding matrix are devoid of fibulin-6, the production of αSMA is reduced resulting in a failure of cells to differentiate from fibroblasts into myofibroblasts after TGF-β stimulation^14^. In this present study we observed that in the absence of fibulin-6, TGF-β mediated actin stress fiber formation is hampered. It is well known that during TGF-β mediated differentiation of fibroblasts to myofibroblasts αSMA is expressed and incorporated into stress fibers. Stress fibers, which already contain β- and γ-cytoplasmic actin gain enhanced contractile force when associated with αSMA, the very reason behind myofibroblasts being heavily contractile compared to fibroblasts^27^. As a consequence, in fibulin-6 deficient cells we found that fibroblast contractility is completely abolished, wherein the cellular networks required for communication during collagen contraction seems to be immensely disturbed. In cardiac tissue it is actually the scaffold created by CF that allows the contraction of the collagen fibers surrounding cardiomyocytes, thus exerting mechanical force to the tissue and myocytes. Thus, our observation with regard to fibulin-6 distribution, spatial occurrence and stress dependent expression in myocardium may suggest that fibulin-6 as an integral and essential component of ECM contributing not only to ECM homeostasis but also governs fibroblast function and activity by regulating TGF-β signaling.

TGF-β signaling orchestrates fibrotic activity of the fibroblast and influences almost every aspect of the remodeling process. On the other hand the ECM proteins critically control the manifestation of TGF-β signaling at every step^28^,^29^, constituting a reciprocal relationship between ECM and TGF-β. Apart from fibrillin-1 and fibronectin, members of the fibulin family have also been reported recently to regulate TGF-β activity in various ways. Studies have shown that regulation of the expression of various fibulin members is mediated via TGF-β. Fibulin-5, which is essential for elastogenesis has been described to be tightly regulated by latent TGF-β binding protein 2 and 4 via its deposition into microfibrils^30^,^31^. Moreover, fibulins are also associated with regulation of TGF-β signaling. For example fibulin-4 is required to bind latent TGF-β binding protein 1 along with fibrillin-1 thus forming a ternary complex, which is indispensible for TGF-β bioavailability to the cells^11^. A study by Zhang H *et al*. has shown that fibulin-2 is crucial for angiotensin–II mediated TGF-β signaling during cardiac hypertrophy^13^. It is important as well for TGF-β mediated effects during MR following myocardial infarction^32^. This suggests that there is a strong association between fibulins and TGF-β signaling. Correspondingly, our current study shows that fibulin-6 deprivation causes a decline in pro-fibrotic effects such as decreased production of collagen and CTGF by nCF, indicating disturbed TGF-β signaling resulting in altered expression of its target genes. Analysis of the SMAD dependent signaling pathway in these cells confirmed that the R-SMAD and co-SMAD failed to translocate into the nucleus following TGF-β stimulus, thus disrupting the signaling events.

As an accepted phenomenon, TGF-β is synthesized as latent precursor complexed with latent TGF-β binding proteins (LTBP). LTBP/TGF-β remains an inactive complex in the matrix until extracellular proteolytic cleavage of LTBP^33^. It is only fibulin-4 whose role has been described in detail in regulating the bioavailability of active TGF-β to the cells from its latent form^11^. For other fibulins, the underlying mechanism in regulating TGF-β activity is still not clear. In our study the question of making latent TGF-β available for cells to trigger signaling via fibulin-6 is excluded as cells were always treated with active form of TGF-β for stimulation. This means that the absence of fibulin-6 influences TGF-β activity either by inhibiting the binding of TGF-β to the corresponding receptors or by blocking the expression of the receptors or by preventing the recruitment and association of the TGF-β receptors to undergo activation and trigger further downstream signaling. We did not identify any change in the appearance of TGFRII and TGFRI on the cell surface of fibulin-6 KD cells compared to wild type. However, there was a prominent decrease in the association of TGFRI with TGFRII when the proteins were pulled down. To confirm this notion we demonstrated that phosphorylation of TGFRI at Ser165 was attenuated by fibulin 6 KD compared to wild type cells. This finding proves that fibulin-6 directly affects TGF-β receptor association and activation to trigger the signaling pathway.

However, apart form the classical SMAD pathway, there are certain non-canonical pathways as well that are independent of SMAD protein activation. TGF-β also directly influences these non-SMAD pathways, which majorly includes various branches of MAP kinase pathways and phosphatidylinositol-3-kinase/AKT pathways, to name only a few. An increase of phospho Akt levels upon TGF-β treatment was comparable in wt and fibulin-6 KD nCF. However very intriguingly we observed constitutive increase of pERK in fibulin-6 KD nCF compared to wt even without TGF-β stimulus, which means that the lack of fibulin-6 invariably activates Ras-Raf-MAPK-ERK pathway and this might not necessarily be via EGFR signaling receptors. This finding didn’t appear as a surprise because many previous publications showed that members of fibulin family are involved in activating ERK pathway, a leading cause for cancer progression. Fibulin-3 for instance is known to suppress ERK pathway via its RGD motif and it has been published that in breast cancer fibulin-3 is downregulated leading to increased ERK and MMP-7 expression^34^. A similar effect was shown for fibulin-3 in lung cancer^35^. A germline KO mice for fibulin-4 shows upregulated pERK 1/2 during aortic aneurysm progression^36^. Also fibulin-1 was shown to inhibit Erk activation when overexpressed in human cancer cell line^37^. Thus, probably it is a general property of members of fibulin family that they suppress MAPK/ERK pathway and when are silenced triggers the pathway for activation. However, how fibulins employ this effect is not known yet. Moreover the activation of MAPK/ERK pathway doesn’t solely occur via EGFR receptors but may also be through Trk A/B, FGFR, PDGFR receptors etc. and we still are unaware whether the activation of the pathway in such instances occur via the EGFR receptors or via other receptors. At this point we also speculate that the activated Ras pathway might also promote the inhibition of TGF-β signaling by impeding nuclear accumulation of SMAD2 and SAMD3 as it has been reported previously^38^,^39^. Thus, it could be an additive effect where in addition to the hindrance in TGFR dimerization and activation leading to SMAD inactivity, increased pERK in fibulin-6 deficient cells may also contribute to reinforce the effect. Based on our observations we hypothesize that mechanistically in the absence of fibulin-6 in the ECM, TGF-β mediated association and activation following phosphorylation of the TGF-β receptors is not possible. This further prohibits SMAD phosphorylation and integration of R-SMADs with co-SMADs to translocate to the nucleus and initiate transcription of essential SMAD-dependent genes required during MR (Suppl. Fig. 3).

However, there are certain limitations to our study. We have not addressed whether fibulin-6 directly or indirectly affect the association of the receptors. How proteins interact specifically with fibulin-6 to affect TGF-β signaling therefore remains an open question. Interaction studies of fibulin-6 with other ECM proteins that are involved in TGF-β signaling need to be performed to address this question. Moreover, lack of viable fibulin-6 knockout mice due to embryonic lethality restrict us to confirm the cardiac phenotype of wound healing and MR following infarction or cardiac hypertrophy in mice rendering conclusions with relevance to clinical questions impossible. Thus, we are in the process to develop strategies for generation of cardio-specific conditional fibulin-6 knockout mice. We have used neonatal CF. Since the expression of fibulin-6 is different during development compared to adulthood we cannot fully exclude that the model chosen affected our findings despite the fact that we have previously shown a prominent role of fibulin-6 fibroblasts in adult mice and heart failure patients.

In summary, we have elucidated the role of fibulin-6 in nCF contractile activity by regulating actin stress fiber formation under the influence of TGF-β. Since cardiac fibroblast contractility is one of the quintessential phenomena during wound healing, fibulin-6 is thus speculated to affect the process of MR. In this paper we have demonstrated a possible mechanism that fibulin-6 may regulate TGF-β signaling by mediating the association and activation of TGF-β receptors for triggering the signaling pathway.

## Materials and Methods

All methods were performed in accordance with the relevant guidelines and local regulations and were approved by the regional authorities (LAVES) for animal care.

### Isolation and culture of neonatal mouse ventricular cardiac fibroblasts

nCF were isolated using the crude method of collagenase type II enzymatic digestion^40^. The final cell suspension constituting cardiomyocytes and fibroblasts was separated by allowing the cell suspension to sediment in an uncoated culture dish at 37 °C for 1 hour. Pre-plating facilitates fibroblasts to adhere to the culture dish separating the cardiomyocytes floating in the cell suspension. nCF were then maintained in Dulbecco’s modified Eagle’s medium (DMEM) high glucose (4.5 mg/ml) with 10% fetal bovine serum (FBS). They were passaged once or twice. A 3T3 cell line of CF was established from nCF using published protocols^41^.

### siRNA transfection

Fibulin-6 silencer select siRNA was synthesized by Ambion Life technologies (s233951, Ambion, Darmstadt, Germany). As negative control, scrambled (scr) siRNA was used (Silencer Select negative control siRNA, Ambion, Darmstadt, Germany). 3T3 or nCF were transfected with 20 nM siRNA via reverse transfection using Lipofectamine RNAiMAX (Invitrogen, Life Technologies, Darmstadt, Germany) as described^14^. The efficiency and specificity of fibulin-6 siRNA has been tested previously^14^.

### Stimulation of nCF

1 million nCF per well were plated in a 6 well plate and the serum content of the media was gradually replaced with 5% serum to 1X Insulin Transferrin Selenium-A (Gibco, Life Technologies, Darmstadt, Germany) for 2 days, then stimulation with 10 nM mouse TGF-β1 (Cell signaling Technology, Inc., Frankfurt, Germany), 23 μM LPA (Biomol, Hamburg, Germany) or 10 ng/ml mouse TNF-α (Cell signaling Technology, Inc., Frankfurt, Germany), was induced for either 15 minutes, 24 hours or 48 hours depending on the experimental requirements.

### Collagen contraction assay

3T3 CF were reversely transfected with respective siRNAs and after 24 hours of transfection collagen gels were prepared. Gels containing 1 mg/ml Cultrex^®^ 3-D Culture Matrix Rat Collagen I (Trevigen, Gaithersburg, MD, USA), 10 X DMEM (Biochrom, Berlin, Germany) and 1 * 10^6^ of trypsinized 3T3 CF, resuspended in DMEM high glucose +10% FBS were mixed on ice. Before the cells were added to the mix, pH of DMEM was adjusted with 2 M NaOH until the color of DMEM changed from yellow to pink. The collagen-cell suspension was then plated into low attachment 24 well plate (Greiner bio-one, Kremsmünster, Austria). After 4 hours of incubation at 37 °C the edges of the gel were loosened from the borders of the plate with a pipette tip. 500 μl of DMEM +2% FBS was then added on top of the gel. For TGF-β1 stimulation 10 nM mouse TGF-β1 was added. After 48 hours the gels were photographed with a digital camera and the area of the contracted gels was measured by using ImageJ (1.47 V, NIH) software.

### Quantitative real-time PCR

Using TRItidy reagent (Applichem, Darmstadt, Germany), total RNA was extracted from nCF. Primer and reagents were purchased from Eurogentec (Cologne, Germany). 500 ng of total RNA was applied for reverse transcription using Reverse Transcriptase Core kit according to the manufacturer’s protocol. Sequences of primer-pairs were as follows: mouse Collagen I forward, 5′-GAG AAC CTG GAA AGG CTG GAG A-3′; mouse Collagen I reverse, 5′-GAT GGT CTG GGT TCA GGT TGG A-3′; mouse CTGF forward, 5′-AGA-CAT-GGC-GTA-AAG-CCA-GGA-AGT-3′; mouse CTGF reverse, 5′-TGT CTC GA ACC AGT GTC TGA GGT-3′; mouse HPRT forward, 5′-TGA TCA GTC AAC GGG GGA CAT A-3′; mouse HPRT reverse, 5′-GCC TGT ATC CAA CAC TTC GAG A-3′. All samples were run in duplicates on a real-time RT-PCR cycler (Rotogene 3000, Corbett Life Science, Hilden, Germany) using SYBR-GREEN (SensiFAST ^TM^ SYBR, Bioline GmbH, Germany) and normalized to HPRT gene expression. Data are expressed as 2^−ΔΔCT^.

### Immunofluorescence staining

Immunofluorescence staining was performed using standard protocols^14^. Cells grown on coverslips or in collagen gels were fixed in 4% paraformaldehyde and permeabilized with 0.2% TritonX-100. After washing cells were blocked with 1% BSA and stained as per experimental requirement with Alexa Fluor 488 Phalloidin (A12379, 1:40, Invitrogen), SMAD3 (51-1500, 1:250, Invitrogen) and DAPI (A1001,0010, 1 μg/μl, Applichem) for nuclear staining. Mounting was done in fluorescent mounting medium (S3023, DAKO). Fluorescence micrographs were captured using Q-capturePro (QImaging, Surrey, Canada), a fluorescence imaging software driving a Retiga CCD camera (QImaging, Surrey, Canada) mounted on an Olympus IX81 microscope (Olympus Europa Holding GmbH, Hamburg, Germany).

### Quantification of stress fibers

The difference in stress fiber phenotype was quantified by measuring the number of stress fibers as described previously^42^. Briefly, Image J (1.47 V, NIH) software was used to generate line profiles for cells within a high-power field. Respective graphs for the line profile are shown in Fig. 1d, representing the distance across the cell on x-axis, and levels of fluorescence on *y*-axis. Each immunofluorescence intensity spike represents an individual stress fiber crossed by the line drawn. Randomly six cells and three regions in each cell for quantification were selected. All the images were obtained with fixed acquisition to ensure analytical consistency.

### Preparation of nuclear and cytoplasmic cell lysate and Western blotting

Whole cell protein lysates of nCF were prepared using RIPA buffer including protease and phosphatase inhibitors (complete mini, no. 11836170001 and PhosSTOP, no.04906845001, Roche Diagnostics, Penzberg, Germany). For nuclear extract preparation, cells were scraped and centrifuged to remove the supernatant, resuspended in ice cold PBS and again centrifuged at 6000 rpm for 5 minutes at 4 °C. The pellet was dissolved in (5 X to the pellet) cytoplasmic extraction buffer (HEPES: 10 nM pH7.9, KCl: 10 mM, EDTA: 0.1 mM, NP-40: 0.3%, protease inhibitor), incubated on ice for 5 minutes with gentle vortexing from time to time, centrifuged at 3000 rpm for 5 minutes and harvested as the cytoplasmic extract. The pellet was resuspended in cytoplasmic extraction buffer (without NP-40) and centrifuged for 5 minutes at 3000 rpm. This washing step was repeated twice. To the pellet equal volume of nuclear extraction buffer (HEPES: 20 mM pH 7.9, KCl: 10 mM, EDTA: 0.1 mM, NP-40: 0.3%, protease inhibitor) was added, resuspended and incubated for 10 minutes on ice, centrifuged at 14000 rpm for 10 minutes at 4 °C and harvested as nuclear extract. Cell lysates (10 μg) were resolved by SDS-PAGE followed by western blot using the following primary antibodies: Collagen I (AB765P, Millipore), CTGF (PA1-22376, Thermo Scientific), SMAD3 (51–1500, Invitrogen), TGFRI (MAB5871, R&D), TGFRI-pSer165 (#12388, SAB) and SMAD4 (#9515), pERK1/2 (#4370), ERK1/2 (#4695), pAKT (#4058), AKT (#9272) all from Cell Signalling. Horseradish-peroxidase-coupled rabbit, mouse or rat IgGs were used as secondary antibodies. Western blots were analyzed on a ChemiSmart 5100 station (Vilbert Lourmat, Eberhardzell, Germany) by using Amersham ECL Plus Detection System (GE Healthcare, Braunschweig, Germany). Density of bands were quantified using Bio1D software (Vilber Lourmat, Eberhardzell, Germany). Membranes were re-probed with anti-GAPDH (#2118, 1:5000, Cell Signalling) and anti-Histone 3 (sc10809, 1:500, Santa Cruz) to confirm equal loading.

### Proliferation Assay

Cell proliferation was assessed using a Colorimetric Cell Viability Kit I (WST-8) from PromoCell (Heidelberg, Germany) according to the manufacturer’s protocol. Briefly, nCF were reverse transfected with scr-siRNA or fibulin-6 siRNA. 5000 cells per well were seeded in a 96 well plate. 24 or 48 hours later the dye CCVKI was added to the cells, incubated for 3 hours and the absorbance at 450 nm measured in a microplate reader (Tecan Sunrise, Männedorf, Switzerland).

### FACS analysis

nCF previously transfected with either fibulin-6- or scr-siRNA were detached from the culture dish by accutase (Sigma, Taufkirchen, Germany) and adjusted to 5 × 10^5^ cells per ml PBS containing 0,1% BSA, 1 mM CaCl_2_ and 1 mM MgCl_2_. Cells were stained with TGFRI (MAB5871, 1:10, R&D) and TRGRII (AF352, 1:10, R&D). Cy2-coupled rat or goat IgGs were used as secondary antibodies respectively. Exclusion of non-viable cells was achieved by staining with 1 μg/ml propidium iodide (Sigma, Taufkirchen, Germany).

Mean fluorescence of samples was measured with Cytomics FC500 FACS (Beckham Coulter, Krefeld, Germany) and analyzed using FlowJo 10-0-8 (FlowJo, Ashland, OR, USA).

### Co-immunoprecipitation of TGFRII and TGFRI

Membrane extracts from fibulin-6- or scr-siRNA-transfected nCF stimulated for 24 hours with 10 nM mouse TGF-β1 were prepared using membrane extraction buffer (20 mM Tris-HCl, pH 7, 4; 1% Trition; 10% Glycerin; 1 mM EDTA) containing protease and phosphatase inhibitors (complete mini, no. 11836170001 and PhosSTOP, no.04906845001, Roche Diagnostics). 1 μg anti-TGFRII (AF352, R&D) or 1 μg goat IgG (I 5000, Vector labs) was covalently crosslinked with 3 mM bis(sulfosuccinimidyl)-suberate (BS^3^;Thermo Scientific, Darmstadt, Germany) to 50 μl protein G dynabeads (Invitrogen, Life Technologies, Darmstadt, Germany). After washing with membrane extraction buffer antibody-protein G dynabead-complexes were incubated with 100 μg membrane extracts for 1 hour at room temperature. The complexes were washed and resuspended in 1× reducing sample buffer, boiled for 5 min and analyzed by western blot using anti-TGFRI (MAB5871, R&D) for detection.

### Statistical analysis

For all statistical analysis GraphPad Prism software Version 5.0d was used (GraphPad Inc, San Diego, CA, USA). Data are presented as median (horizontal bar) and interquartile (box) and 5/95% (whiskers) range. If data showed non-Gaussian distributions or significantly different SDs, Kruskal–Wallis test followed by Mann–Whitney U test for group wise comparisons with Holm’s correction for multiple testing was employed. All the data represent the mean of at least three independent experiments. P values less than 0.05 are considered statistically significant.

## Additional Information

**How to cite this article**: Chowdhury, A. *et al*. Fibulin-6 regulates pro-fibrotic TGF-β responses in neonatal mouse ventricular cardiac fibroblasts. *Sci. Rep.* **7**, 42725; doi: 10.1038/srep42725 (2017).

**Publisher's note:** Springer Nature remains neutral with regard to jurisdictional claims in published maps and institutional affiliations.

## Supplementary Material

Supplementary Information
