# Supplementary material for: Fibulin-6 regulates pro-fibrotic TGF-β responses in neonatal mouse ventricular cardiac fibroblasts
Source: Sci Rep. 2017 Feb 17;7:42725. doi: 10.1038/srep42725 (PMC5314373; doi:10.1038/srep42725)
Supplement: Supplementary Information [file srep42725-s1.pdf]

Supplementary Information File:

Fibulin-6 regulates pro-fibrotic TGF-β responses in neonatal mouse ventricular cardiac fibroblasts

Arpita Chowdhury[a,b§]; Lisa Hasselbach[a§]; Frank Echtermeyer[a]; Nidhi Jyotsana[c]; Gregor Theilmeier[a,d]; Christine Herzog[a*].

[a]Department of Anesthesiology and Intensive Care Medicine, Hannover Medical School, Hannover, Germany

[b]Department of Cellular Biochemistry, University Medical Center Göttingen, Göttingen.

[c]Department of Hematology, Hemostasis, Oncology and Stem cell Transplantation, Hannover Medical School, Hannover, Germany

[d]Perioperative Inflammation and Infection, Department of Human Medicine, Faculty of Medicine and Health Sciences, University of Oldenburg, Oldenburg, Germany

Supplementary figures:

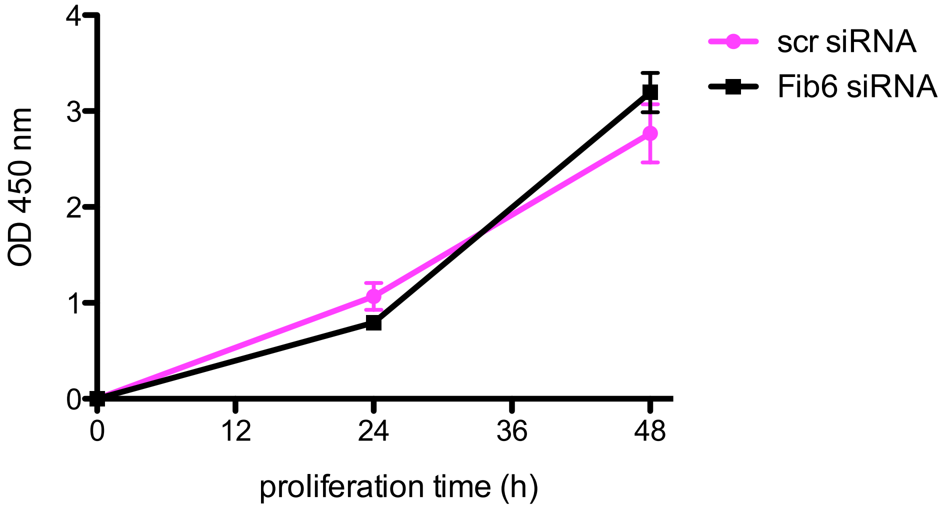

Supplementary Fig. 1

Cellular proliferation rate in fibulin-6 KD cells.

Cell proliferation was assessed in a colorimetric assay using the tetrazolium salt WST-8. nCF were transfected with scr siRNA and fibulin-6 siRNA and 24h and 48h after transfection the number of viable cells were measured at OD 450 nm. scr siRNA and fibulin-6 siRNA -transfectecd nCF show after 24h and 48h no differences in cell proliferation (n=5, p=ns).

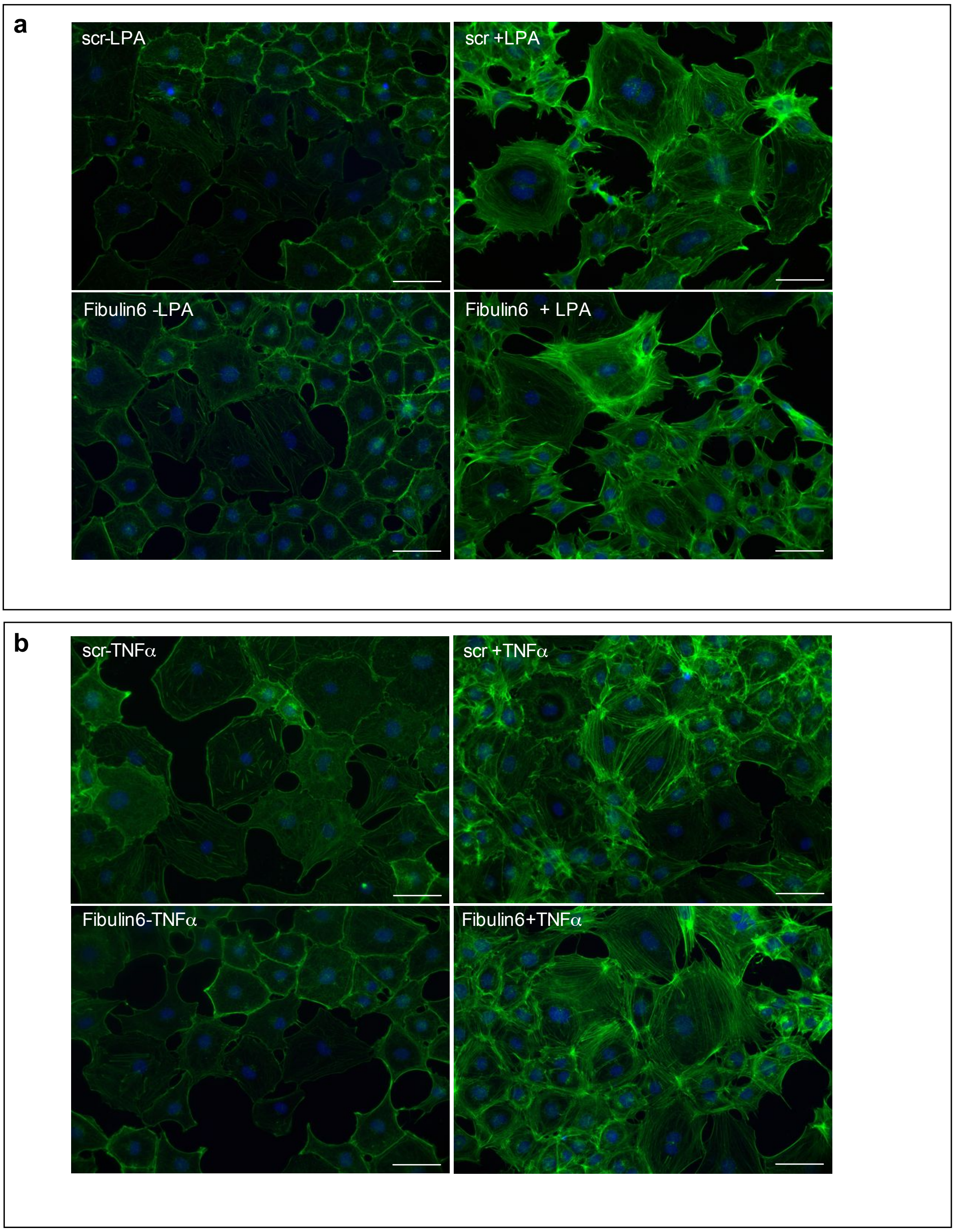

Supplementary Fig. 2

Effect of fibulin-6 KD on cortical F-actin formation induced by LPA and TNF-α in fibroblasts.

nCF transfected with either scr siRNA or fibulin-6 siRNA were treated with **a)** 23μM LPA or **b)** 10ng/ml TNF-α for 24 hours, both showing an increase in cortical F-actin formation upon treatment compared to non-treated cells in scr

transfected nCF. In fibulin-6 KD cells the treatment results also in cortical F-actin formation without any visible difference in comparison to control cells (scale bar = 100 µm).

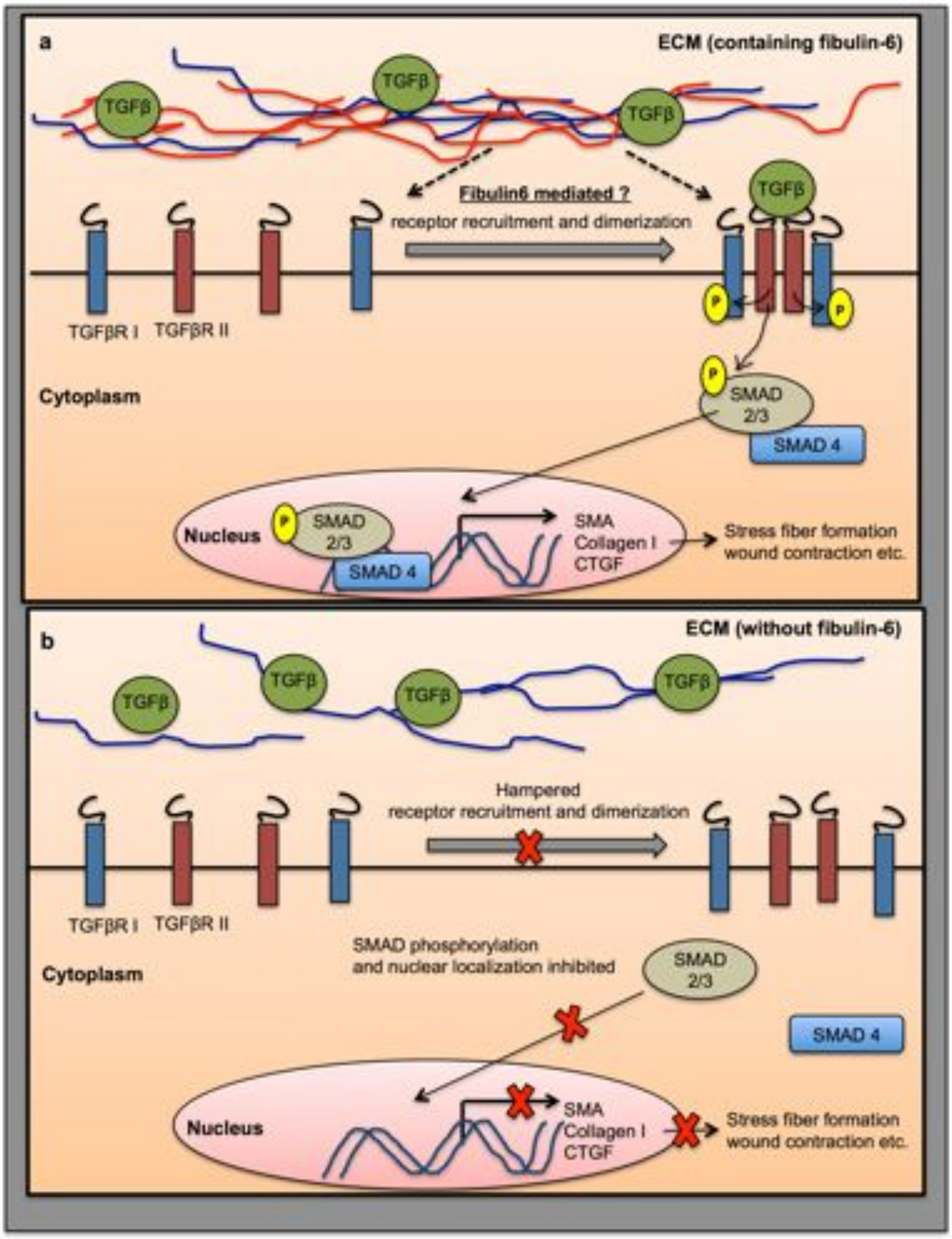

Supplementary Fig. 3

Schematic view of the proposed mechanism of fibulin-6 mediated TGF-β signaling. **(a)** In wild type fibroblasts TGF-β ligand is recognized by the receptor TGFRII. TGFRII further oligomerizes together with TGFRI and activates TGFRI by phosphorylation at Ser 165. TGFRI then phosphorylates R-SMADs (SMAD 2/3), which can now bind with coSMAD (SMAD4). This complex of R-SMAD and co-SMAD translocate inside the nucleus where it act as a transcription factor for the expression of various target genes required for stress fiber formation (αSMA) and wound contraction (Collagen I, CTGF) etc. **(b)** On the other hand in the absence of fibulin-6, receptor dimerization for TGF-β ligand binding is severely obstructed. As a result TGFRI is not in a close proximity to get activated via phosphorylation by TGFRII. Consequently further downstream activation of SMAD proteins are also blocked. Ultimately the SMAD proteins are not translocated into the nucleus for transcribing genes required for wound contraction and healing.
